# Impact after the Change from Voluntary to Universal Oral Rotavirus Vaccination on Consecutive Emergency Department Visits for Acute Gastroenteritis among Children in Kobe City, Japan (2016–2022)

**DOI:** 10.3390/vaccines10111831

**Published:** 2022-10-29

**Authors:** Hiroshi Yamaguchi, Kandai Nozu, Hiroaki Hanafusa, Yoshinori Nambu, Takumi Kido, Atsushi Kondo, Akihiro Tamura, Hiroyuki Awano, Ichiro Morioka, Hiroaki Nagase, Akihito Ishida

**Affiliations:** 1 Department of Pediatrics, Kobe University Graduate School of Medicine, 7-5-2 Kusunoki-cho, Chuo-ku, Kobe 650-0017, Japan; 2 Department of Pediatrics and Child Health, Nihon University School of Medicine, 30-1 Oyaguchi, Kami-cho, Itabashi-ku, Tokyo 173-8610, Japan; 3 Kobe Children’s Primary Emergency Medical Center, 1-4-1 Wakihamakaigandori, Chuo-ku, Kobe 651-0073, Japan

**Keywords:** children, emergency department, gastroenteritis, observational study, rotavirus, norovirus, vaccine

## Abstract

Rotavirus (RV) is the leading cause of acute gastroenteritis (AGE), particularly in infants. In 2006, the high efficacy of oral RV vaccines (RVVs, Rotarix^TM^ and RotaTeq^TM^) was demonstrated. Voluntary RVV started in Japan in 2011, and in October 2020 were launched as universal oral RVVs in Japan. However, the impact of changes from voluntary to universal RVVs has not been studied in a primary emergency medical center in Japan. We investigated changes in the number of pediatric patients with AGE after introducing universal RVVs in our center. A clinical database of consecutive patients aged <16 who presented to Kobe Children’s Primary Emergency Medical Center between 1 April 2016 and 30 June 2022 was reviewed. After implementing universal RVVs, fewer children presented with RV-associated AGE (the reduction of proportion of the patients in 2022 was −61.7% (all ages), −57.9% (<1 years), −67.8% (1–<3 years), and −61.4% (3–<5 years) compared to 2019). A similar decrease in those of age who were not covered by the universal RVV was observed. There was a significant decline in the number of patients with AGE during the RV season who presented to the emergency department after implementing universal RVVs.

## 1. Introduction

Rotavirus (RV) is the leading cause of acute gastroenteritis (AGE), particularly in infants and young children. Before the introduction of RV vaccines (RVVs), RV infection was the primary cause of severe AGE in young children and a substantial cause of hospitalization or death worldwide, especially in developing countries [1,2,3,4]. Currently, safe and effective oral RVVs are available [5]. According to the ROTA Council, 114 countries have introduced RVVs, and 57% of infants lived in countries where RVVs will be introduced by March 2022 (ROTAVIRUS VACCINE INTRODUCTION AND COVERAGE; https://preventrotavirus.org/resources/rotavirus-disease-and-vaccines-series-of-briefs-2022/, accessed on 10 July 2022). In addition, studies in many countries have reported significant decreases in RV hospitalization and mortality after the introduction of RVVs [5,6,7,8,9].

In 2006, the high efficacy of oral RVVs (Rotarix™ [RV1]: GlaxoSmithKline Biologicals, Rixensart, Belgium, and RotaTeq^TM^ [RV5]; Merck & Co., Kenilworth, NJ, USA) was demonstrated [10,11]. The World Health Organization (WHO) recommended the inclusion of RVVs in national immunization programs (NIPs) worldwide in 2009 [12]. In Japan, RVVs were available since 2011 but were not included in the NIP until 2020. Two voluntary vaccines (Rotarix^TM^ and RotaTeq^TM^) were launched in Japan in November 2011 and July 2012, respectively. The estimated voluntary RVV coverage rates in Hyogo Prefecture, to which Kobe City belongs, were 1% in 2011, 25% in 2012, 42% in 2013, and 49% in 2014 [13]. Although there is no recent data for Hyogo Prefecture, there is a report that the vaccine coverage rate increased from 30.0% in 2012 to 78.4% in 2019 after the implementation of voluntary RVVs in Japan [5].

Rotarix^TM^ is a live attenuated monovalent human RVV derived from a single-strain human RV with the G1P [8] genotype [10]. In contrast, RotaTeq^TM^ is a pentavalent human-bovine reassortant containing a mixture of five reassortants carrying the genes encoding human G1, G2, G4, and P [8] in a genetic background of the bovine WC3 RV (G6P7 [5]) [11]. Previous studies have shown a decline in severe pediatric RV-AGE after the introduction of voluntary RVV in Japan [14,15]. In Japan, the highest prevalence of RV and norovirus (NV) occurs between March–June and November–February, respectively; voluntary RVVs have led to a significant reduction in the number of pediatric patients (≤5 years of age) with AGE at our primary emergency medical center [13]. In October 2020, Rotarix^TM^ and RotaTeq^TM^ were launched in the NIP in Japan. Patients are free to choose which vaccines to use—generally, the vaccine that is introduced by the hospital where the patient visits are administered. Babies born in August 2020 or later were included in NIP. The first inoculation was administered from 6 to <15 weeks after birth. Subsequent inoculations included an additional dose of Rotarix ^TM^ and two additional doses of RotaTeq^TM^ and were administered at intervals of 27 days or more. Despite the effectiveness of such vaccines, reports on the effect of universal RVVs on the number of patients with AGE in primary emergencies are scarce, and the impact of changes from voluntary to universal RVVs has not yet been studied in children’s primary emergency medical centers in Japan.

This study aimed to investigate the changes in the number of patients with AGE after switching the RV vaccination scheme from voluntary to universal in Kobe Children’s Primary Emergency Medical Center in Japan, especially during the norovirus (NV)- and RV-prevalent seasons.

## 2. Materials and Methods

### 2.1. Study Design, Patients, and Definition

This retrospective, clinical observation time-series study was conducted with the approval of the Ethics Committee of the Kobe Pediatric Primary Emergency Medical Center. The need for informed consent was waived due to the retrospective nature of the investigation and its accessibility to the public using our website. To determine the impact of changes from voluntary to universal RVVs, we reviewed the clinical database of consecutive patients with AGE who were younger than 16 years and presented to the emergency department (ED) of Kobe Children’s Primary Emergency Medical Center between 1 April 2016, and 30 June 2022. Our study began on 1 April 2016 because our center had begun to ask all caregivers of patients who visited our center for a history of vaccines using a questionnaire. The questionnaire only stated whether the vaccine was administered and did not include the number of vaccinations. The questionnaire included questions on RV, hepatitis B, *Haemophilus influenzae* type b, *Streptococcus pneumoniae*, DPT-IPV (diphtheria, pertussis, tetanus, polio), BCG, MR (measles & rubella), varicella, mumps, Japanese encephalitis, and palivizumab (respiratory syncytial virus). However, history was not available from July to December 2016 for technical reasons.

Kobe is in the southern part of Japan’s main island, with approximately 1.5 million residents, 200,000 of whom are children under the age of 16. Our medical center has the largest primary ED in Kobe City, and provides medical services for pediatric patients without trauma, only during holidays and outside regular working hours (7:30 p.m.–7:00 a.m.).

AGE diagnosis was clinically determined by pediatricians in the ED. Pediatricians chose disease diagnoses linked to ICD-10 codes. Rapid antigen tests for RV and NV are available but are not used in ED settings because they do not contribute to the change of the course of treatment. Therefore, the specific virus in patients with AGE is unknown in this study. Patients with AGE were identified from a medical database based on their AGE diagnosis. The inclusion criteria were as follows: cases of viral AGE (viral AGE that was clinically diagnosed) from the database, excluding bacterial AGE, allergic AGE, acute vomiting or diarrhea, simple gastritis, and neonatal vomiting. Acute diarrhea was excluded because it could be due to various differential diagnoses, including endocrine disease (e.g., hyperthyroidism), inflammatory disease (e.g., inflammatory bowel disease), malabsorption syndrome, irritable bowel syndrome, and medicinal side effects.

The RV- and NV-prevalent seasons were defined as the seasons from March to June and November to February, respectively, based on our previous study [13]. In Japan, the coronavirus disease 2019 (COVID-19) pandemic spread the same year the universal vaccine program began. Elementary, junior high, and high schools were closed on 3 March 2020, and the Japanese government recommended that people stay home. The Japanese government issued an emergency declaration on 9 April 2020. The declaration was lifted on 25 May 2020, and schools re-opened on 1 June 2020. It was not mandatory to close nursery schools and kindergartens; however, most of these institutions were also closed during this period. In 2020, the number of patients who visited our center differed from the usual year because of the closure of schools under the emergency declaration. Therefore, changes in the number of patients with AGE were compared, except during the COVID-19 era, which is defined as the period from March 2020 to February 2021.

We further extracted patient data under the ages of 1, 1–<3 years, and 3–<5 years who were affected more by severe symptoms [4] in a subgroup analysis and compared the changes in the number of patients with AGE.

The annual population of Kobe City under the age of 16 was retrieved from the homepage website of Kobe City (https://www.city.kobe.lg.jp/a47946/shise/toke/toukei/jinkou/index.html (10 July 2022)).

### 2.2. Statistical Analysis

The results are expressed as number or number (%) or median (interquartile range [IQR]). Statistical analysis was performed on the number of patients with AGE before and after NIP and adjusted for population. The total number of patients with AGE in the analyzed years divided by the number of years used was defined as “AGE+”; the total population divided by the number of years analyzed, subtracted by the “AGE+” was defined as “AGE-.” The data were analyzed using the chi-square test. All analyses were performed using GraphPad Prism 5.0 (GraphPad Software, San Diego, CA, USA). Statistical significance was set at *p* < 0.05.

## 3. Results

### 3.1. Patient Demographics

During the study period, 137,651 pediatric patients visited our center. Of whom, 21,519 (15.6%) were clinically diagnosed with AGE. The median age was 4.3 years (IQR; 2.1–7.3); 53.7% were males (n = 11680) and 45.7% were females (n = 9839).

### 3.2. Monthly Changes in the Number of Patients with Gastroenteritis Stratified into <1 Year, 1 to <3 Years, 3 to <5 Years, and ≥5 Years

**Figure 1:**
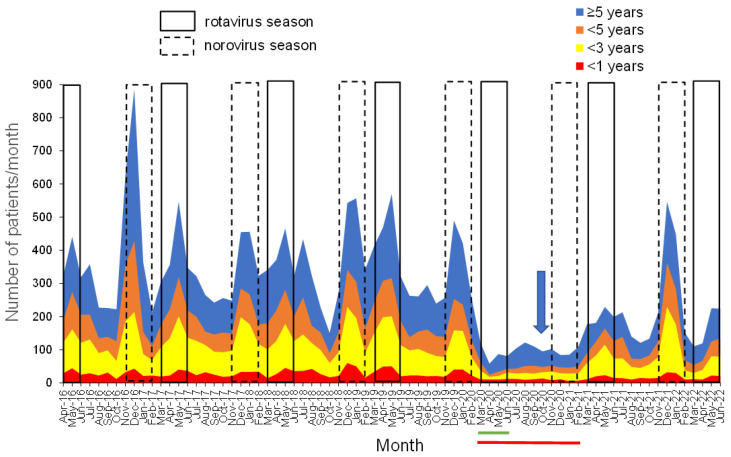
Monthly changes in the number of patients with AGE stratified into <1 year, 1 to <3 years, 3 to <5 years, and ≥5 years. The figure shows the monthly changes in the number of patients with AGE (<1-year-old (red); 1–<3-year-old (yellow); 3–<5-year-old (orange); and ≥5-year-old (blue)). The vertical axis indicates the number of patients with AGE. The horizontal axis indicates calendar months. The blue arrow indicates the date of the implementation of universal RVV. The green bar under the graph indicates the period of the state of emergency. The red bar under the graph indicates the period of the COVID-19 era. AGE, acute gastroenteritis.

Figure 1 shows the monthly changes in the number of patients with AGE who visited our center. The number of patients with AGE was bimodal during the RV season (spring) and NV season (winter) throughout the year. The national universal NIP for RVVs was implemented in October 2020 (blue arrow; Figure 1). Until 2019, the peak number of patients with AGE for all ages at our center during the RV and NV seasons was approximately 600–800 and 600–1100, respectively. The respective peak numbers of patients with AGE at our center during the RV and NV seasons before COVID-19 were approximately 40–50 and 30–60 for ages <1 year; 110–160 and 110–170 for ages 1–<3 years; 100–120 and 90–220 for ages 3–<5 years; and 160–260 and 180–470 for ages ≥5 years. However, during the 2020 COVID-19 pandemic, the number of patients with AGE remarkably decreased, and no bimodal peaks of patients with AGE during RV and NV seasons were observed (red bar in Figure 1). In 2021, the number of patients with AGE for all ages and all stratified ages increased slightly during the RV season, although lower than the voluntary RVV era, and the number of patients with AGE during the NV season highly increased, similar to the pre-COVID-19 era (2020). In 2022, compared to the voluntary RVV era, the peak number of patients with AGE during the RV season apparently decreased.

### 3.3. Annual Number of Patients with AGE during RV and NV Seasons and Their Vaccination History

Table 1 shows the annual numbers of patients with AGE during the RV and NV seasons and their vaccination history at susceptible age (<5-year-old). Vaccine questionnaires were given to and collected from all caregivers; the vaccine status was “known” in 96.5% and “unknown” in 4.5%. Of the total 662 patients aged <1 year during the RV season, 377 had a history of RVV, 253 had no history of RVV, and 32 were unknown. Of the 2180 patients aged 1 to under 3 years, 1035 had a history of RVV, 1078 had no history of RVV, and 67 were unknown. Of the 1743 patients aged 3 to under 5 years, 710 had a history of RVV, 965 had no history of RVV, and 68 were unknown. Excluding the COVID-19 era in 2020, there was an increase in the number of patients with AGE, who are 5 years old and below, and received the RVV from 2021 onwards.

The total number of patients who visited our center decreased by approximately 50% after the COVID-19 era (Table 1). However, the total number of patients with AGE during the NV season in 2021–2022 was not different from that in the pre-COVID-19 era; the number of patients aged 1 to <5 years increased in 2021–2022 compared to that in 2019–2020 (Table 1). In contrast, after implementing the RVV NIP in October 2020, the number of patients with AGE during the RV seasons apparently decreased (Table 1). The reduction of proportion of the patients in 2022 was −61.7% (all ages), −57.9% (<1 years), −67.8% (1–<3 years), and −61.4% (3–< 5 years) compared to that in 2019 (all ages: 1772 (2019) vs. 678 (2022), <1 year-old: 152 (2019) vs. 69 (2022), 1–<3 years: 510 (2019) vs. 164 (2022), 3–<5 years: 391 (2019) vs. 154 (2022)). Table 2 shows the annual population of Kobe City under 16 years. From 2016 to 2022, the population of those under 16 years continued to decline, and the population in 2022 decreased by 7.6% compared to that in 2016. This decrease was minor compared to the decrease in the number of patients with AGE during the RV season.

**Figure 2:**
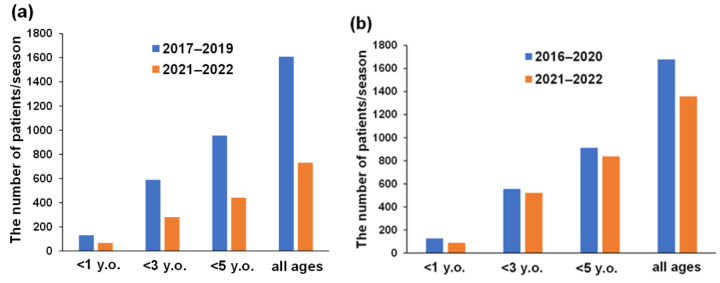
The number of patients aged < 1 year, 1–<3 years, 3–< 5 years, and all ages per RV and NV seasons pre- and post-RVV NIP. The number of patients aged <1 year, 1–<3 years, 3–<5 years, and ≥3 years all ages per (**a**) RV seasons and (**b**) NV seasons pre- and post-RVV NIP. The vertical axis indicates the number of patients with AGE per season ((**a**) the number of patients with AGE divided by three (2017, 2018, and 2019) and two (2020 and 2021); (**b**) the number of patients with AGE divided by four (2016–2017, 2017–2018, 2018–2019, and 2019–2020) and the data for 2020–2021 was not divided as it is a single year). RV, rotavirus; NV, norovirus; RVV, rotavirus vaccine; NIP, national immunization program; AGE, acute gastroenteritis.

Figure 2 shows the number of patients aged <1 year, 1–<3 years, 3–<5 years, and all ages per RV and NV seasons of pre- (voluntary RVV) and post-RVV NIP. During the RV seasons, reductions of 48.5%, 53.8%, 55.7%, and 54.5% were observed in patients aged <1 year, 1–<3 years, 3–<5 years, and all ages, respectively. However, during the NV seasons, children most susceptive of AGE who were aged 1–<3 years increased by 1.1%, although other age groups show reductions of 31.1% (<1 year), 10.9% (3–5<years) and 19% (all ages). When the total number of patients with AGE pre-NIP in the RVV era of 2017–2019 and the number of post-RVV NIP patients from 2021–2022 were adjusted by population and compared using a chi-squared test, the decrease was significant (*p* < 0.0001, *p* < 0.0001, and *p* < 0.0001 (3 years, <5 years, and all ages): 2017–2019 vs. 2021–2022).

### 3.4. Changes in the Number of Patients with AGE According to Birth Year

**Figure 3:**
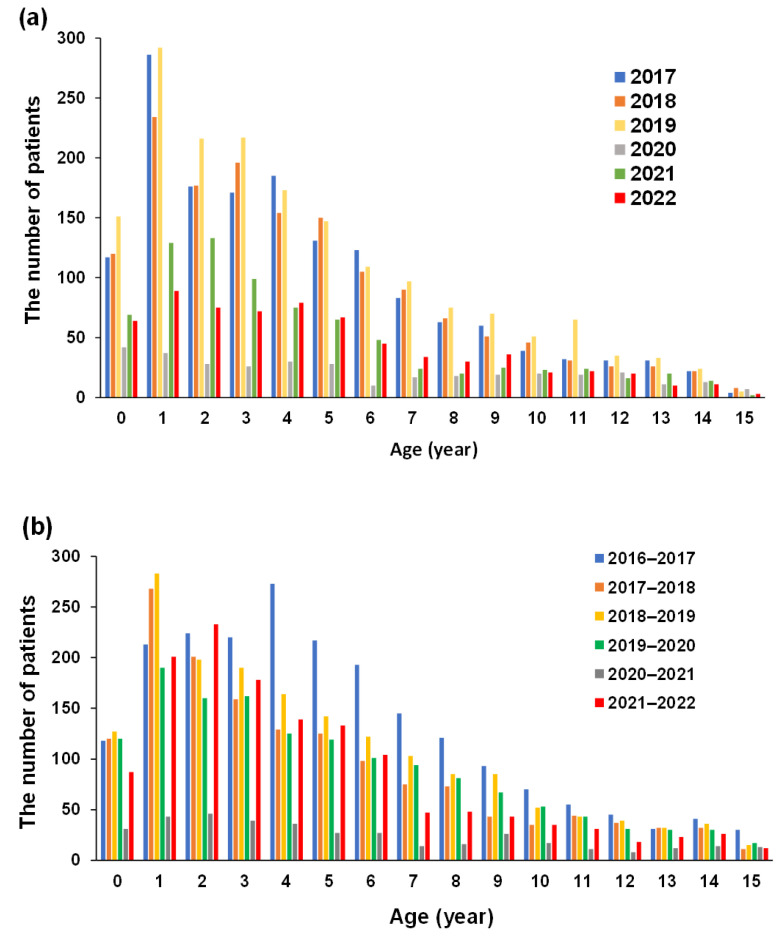
Changes in the annual number of patients with AGE according to age during RV (**a**) and NV (**b**) Scheme 2016 and ended in June 2022, the data for 2016 during the RV season are not shown. AGE, acute gastroenteritis; RV, rotavirus; NV, norovirus.

Figure 3 shows the changes in the number of patients with AGE during the RV and NV seasons according to birth cohort. After implementing the RVV NIP in October 2020, the number of patients of all ages and <3 years old during the RV season decreased in 2021 and further decreased in 2022 compared to pre-RVV NIP (2017–2019). The number of patients with AGE during NV seasons under 6 years old did not change, although the number of patients aged 7 years or older (school ages) tended to decrease.

## 4. Discussion

We conducted a retrospective observational study of the impact of changes from voluntary to universal RVVs by analyzing consecutive patients with AGE who visited our center. We show that after implementing universal RVVs in October 2020, the number of children with AGE who presented to our ED apparently decreased during the RV season.

The present study demonstrates the impact of RVVs and an approximately 60% reduction in the number of pediatric patients with AGE during the RV season compared to the pre- and post-universal vaccine era. The high efficacy of RVVs for RV-associated mortality and severity or reduced hospitalization were reported worldwide [8,16,17]. RVV efficacy is reported to be associated with socio-economic status (SES); in low- (51%), middle- (86%), and high-income groups (93%) [18]. As Japan is a developed country with a high SES, the high impact in the present study is consistent with previous reports [18]. In the present study, we found a similar decrease in the number of older patients with AGE for whom universal RVVs did not cover. Similar herd protection was reported in other studies [19,20]. Considering the significant disease burden of AGE and the cost-effectiveness of RVVs, RVVs are considered very effective [21,22]. After implementing RVVs, some studies reported seasonal changes in the subsidization period to an earlier period for AGE [23]. In this study, seasonal changes were not assessed because evaluations were made only within 21 months after the implementation of RVV in the NIP.

After the implementation of voluntary RVVs in Japan, the vaccine coverage rate increased from 30.0% in 2012 to 78.4% in 2019 [5]. The precise vaccination coverage rate after implementing universal RVVs has not been reported in Japan. However, the RVV coverage rate is expected to reach >95% throughout Japan after implementing universal RVVs because similar coverage rates were achieved for other routine vaccines [24]. The present study shows that approximately 60% of patients under 3 years old with AGE who visited our center during RV seasons were vaccinated in 2022 (post-universal vaccine era), and the vaccination rates increased by approximately 20% when looking at vaccination history under 3 years. A relatively high percentage of vaccinated children with AGE visited our center. This increased number is probably due to the number of children who had an RVV increase due to universal RVV implementation, although RVVs do not completely protect young children against AGE, they reduce the severity [25]. In addition, there are two other possibilities. The first possible reason is that the main targeted RV strains changed, which were vaccine insensitive. Tsugawa et al. reported trends in GP genotypes of RV over 30 years (1987–2020) in Sapporo City, Japan [5]. Before 2000, G1P [8] was dominant, and before the implementation of voluntary RVVs in Japan from 2001 to 2011, it changed among the five major GP genotypes of RV (G1P [8], G2P [4], G3P [8], G4P [8], and G9P [8]). After 2012, new GP genotypes emerged and became dominant (G1P [8], G3P [8], G8P [8], and G12P [8]). Changes in strains were reported in many countries [5,26,27]. As such strains are vaccine-preventable, there is currently no definitive evidence to suggest the emergence of new RV strains that can escape vaccine immune pressure or emerge from vaccine-independent genotype variation. We could not draw any conclusions as we could not measure RV strains at our center. The second reason is the increased proportion of other causative pathogens of AGE. It is expected that the implementation of the RVV will create certain immune pressure to decrease RV infection but may increase the incidence of AGE by other pathogens [28,29,30,31,32]. In Japan, other pathogens causing AGE were reported, with NV being the most common, followed by RV, adenovirus, sapovirus (SaV), and human astrovirus (HAstV) [33]. HAstV has also been reported in Japan during spring (March–May) [34]. SaV infection has recently increased in the post-RV vaccination era from 2009 to 2019 in Japan (4.3% in the pre-RVV era to 6.1% in the post-vaccine era) [28]. In addition, the prevalence of NV remained stable or increased, whereas that of RV-AGE dramatically decreased, similar to the present study [29,30]. However, because our study could not distinguish between RV-AGE and non-RV-AGE, and as we did not check the RV strains, we could not assess these hypotheses.

RV is a disease that primarily affects young children (<5 years); thus, the impact of RVV would most likely be seen in this age group. Arakaki et al., in a systematic review and meta-analysis, reported the prevalence of RV among older children (>5 years) and adults suffering from diarrhea [35]. They calculated pooled conditional RV prevalence and showed that pooled prevalence of RV among older children and adolescents was 8.7%. Therefore, the numbers of patients with RV AGE in older children and adolescents are probably small. However, the accurate prevalence of RV in older children is unknown because the rapid antigen test for RV is rarely performed in older children, and there were no recent epidemiological studies among older children with RV AGE in Japan. Kyo et al. reported increasing severe RV AGE in children older than 5 years after voluntary RVV introduction in Japan [36]. They warned that children older than 5 years could be affected by severe RV infection and suggested prompt intervention for this age group, similar to that of infants. Therefore, including the data of older children and adolescents is necessary.

COVID-19 was declared a pandemic in 2020 [37]. The first adult case of COVID-19 was reported in Japan on 16 January 2020. Schools were closed on 3 March 2020. The Japanese government issued an emergency declaration on 9 April 2020, which was lifted on 25 May 2020, and school attendance resumed on 1 June 2020. As a precautionary measure, the Japanese refrained from holding face-to-face activities and were encouraged to wear masks and wash their hands. Good hygiene was found to reduce the number of patients. We recently reported the impact of the state of emergency during the COVID-19 pandemic in 2020 on asthma exacerbations (AE) among children in our ED [38]. A spring peak in the number of patients with AE was not observed in 2020, which is similar to the reduction in patients with AGE in the present study [38]. Although the winter peaks (NV seasons) were not observed in the number of patients with AGE, the fall peak of patients with AE was again observed after the state of emergency was lifted [38]. The possible difference in the transition of the number of patients with AE and AGE may be because many Japanese refrained from eating out during the winter of 2020 [39]. Restaurants are at a high risk of NV infection due to frequent contact of hands with food, food sharing, and surfaces such as tables or doorknobs [40].

Our study had several strengths. First, a relatively large number of consecutive patients were included in the study. Notably, we have published various epidemiological studies using big data based on our center’s database [24,38,41,42]. Second, our observational study period was relatively long (7 years). Finally, we obtained the RVV history of most patients (96.5%). Nevertheless, our study had some limitations. First, it is unknown whether our results can be applied to other areas because this study was conducted at a single center. Second, detailed patient backgrounds were unavailable in the database; therefore, we could not obtain data on the severity of the disease or side effects, such as intussusception, and there was no information on whether the patients were transferred to other hospitals. Third, since we generally do not test for AGE in our center, the causative microorganism could not be identified. Fourth, it is unknown which RVV the patient received and how many times the patient was vaccinated. Finally, an emergency was declared following the COVID-19 pandemic within the study period, which complicated the assessment of changes in the number of patients with AGE. Since we have only been able to follow up for 2 years after the post-COVID-19 era, we cannot exclude the impact of COVID-19 on the number of patients with AGE. In addition, measures put in place to control the spread of COVID-19 (hand washing and social distancing, among others) would have impacted other communicable diseases, including RV. With this type of ecological study, we could not adjust for any confounders; thus, there could be other reasons for the decrease in AGE observed over time. Despite these limitations, our data show the impact of RVVs in reducing the number of patients with AGE in the ED.

## 5. Conclusions

Our results show a significant decline in the number of patients with AGE during RV seasons, rather than NV seasons, who presented to the ED after implementing universal RVVs.

## Figures and Tables

**Table 1:** (A) The annual number of patients and vaccination history of patients with acute gastroenteritis who visited our emergency department during rotavirus seasons (March–June). (B) The annual number of patients with acute gastroenteritis who visited our emergency department during norovirus seasons (November–February).

(**A**)								
Year	Total Number of Visits	All Ages	<1 Year	Rotavirus Vaccination (+)	1–<3 Years	Rotavirus Vaccination (+)	3–<5 Years	Rotavirus Vaccination (+)
		Number of Patients	Number of Patients		Number of Patients		Number of Patients	
2016 ^#^	20,490	1084	98 (9.0%)	52	306 (28.2%)	117	265 (24.4%)	67
				(53.1%)		(38.2%)		(25.3%)
2017	28,674	1554	117 (7.5%)	68	462 (29.7%)	184	356 (22.9%)	120
				(58.1%)		(39.8%)		(33.7%)
2018	27,643	1502	120 (8.0%)	67	411 (27.4%)	179	350 (23.3%)	169
				(55.8%)		(43.6%)		(48.3%)
2019	27,066	1772	152 (8.6%)	88	510 (28.8%)	252	391 (22.1%)	174
				(57.9%)		(49.4%)		(44.5%)
2020	13,274	346	42 (12.1%)	20	65 (18.8%)	44	56	18
				(47.6%)		(67.7%)	(16.2%)	(32.1%)
2021	13,291	786	69 (8.8%)	45	262 (33.3%)	152	174 (22.1%)	87
				(65.2%)		(58.0%)		(50.0%)
2022 ^$^	6630	678	64 (9.4%)	37	164 (24.2%)	104	151 (22.3%)	78
				(57.8%)		(63.4%)		(51.7%)
(**B**)								
Year		All Ages		<1 Year		1–<3 Years	3–<5 years	
		Number of Patients		Number of Patients		Number of Patients	Number of Patients	
2016–2017		2089		118 (5.6%)		437 (20.9%)	494 (23.6%)	
2017–2018		1482		120 (8.1%)		469 (31.6%)	288 (19.4%)	
2018–2019		1716		147 (8.6%)		461 (26.9%)	354 (20.6%)	
2019–2020		1423		120 (8.4%)		350 (24.6%)	287 (20.2%)	
2020–2021		380		31 (8.2%)		89 (23.4%)	75 (19.7%)	
2021–2022		1358		87 (6.4%)		434 (32.0%)	317 (23.3%)	

^#^ From 1 April to 31 December 2016. ^$^ From 1 January to 30 June 2022. Data are represented as number or number (%). The proportion of the number of patients was calculated by the number of patients in each category divided by the number of patients of all ages. The proportion of the number of positive RVV was calculated by the number of patients with positive RVV in each category divided by the number of patients in each category.

**Table 2:** Annual population in Kobe City under the age of 16.

Years	Annual Population < 16
2016	207,458
2017	205,085
2018	204,599
2019	201,499
2020	198,494
2021	195,115
2022	191,674

The annual population of Kobe City under the age of 16 years was retrieved from the homepage website of Kobe City (https://www.city.kobe.lg.jp/a47946/shise/toke/toukei/jinkou/index.html (accessed on 10 July 2022).

## Data Availability

The datasets generated during and/or analyzed during the current study are available from the corresponding author upon reasonable request.
